# Supplementary material for: Differential Distribution and Activity Profile of Acylpeptide Hydrolase in the Rat Seminiferous Epithelium
Source: Biomedicines. 2022 Jul 4;10(7):1591. doi: 10.3390/biomedicines10071591 (PMC9312705; doi:10.3390/biomedicines10071591)
Supplement: Supplementary file 1 [file biomedicines-10-01591-s001.zip › biomedicines-1767290-supplementary.pdf]

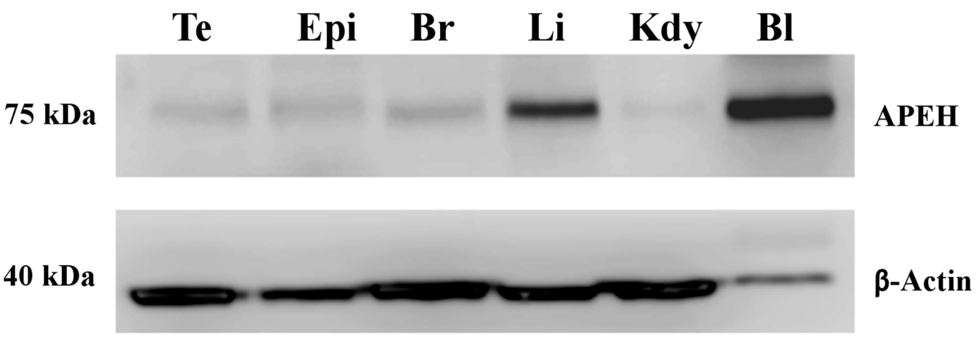

Supplementary Figure S1. APEH levels from different tissues of perfused male rats. (A) Protein extracts from testis (Te), epididymis (Epi), brain (Br), liver (Li), kidney (Kdy) and Blood (Bl) were obtained from male rats. Western blot analysis of these extracts (30 µg/lane) were fractionated by SDS-PAGE, transferred to PDVF membrane and immunoblotted for APEH.

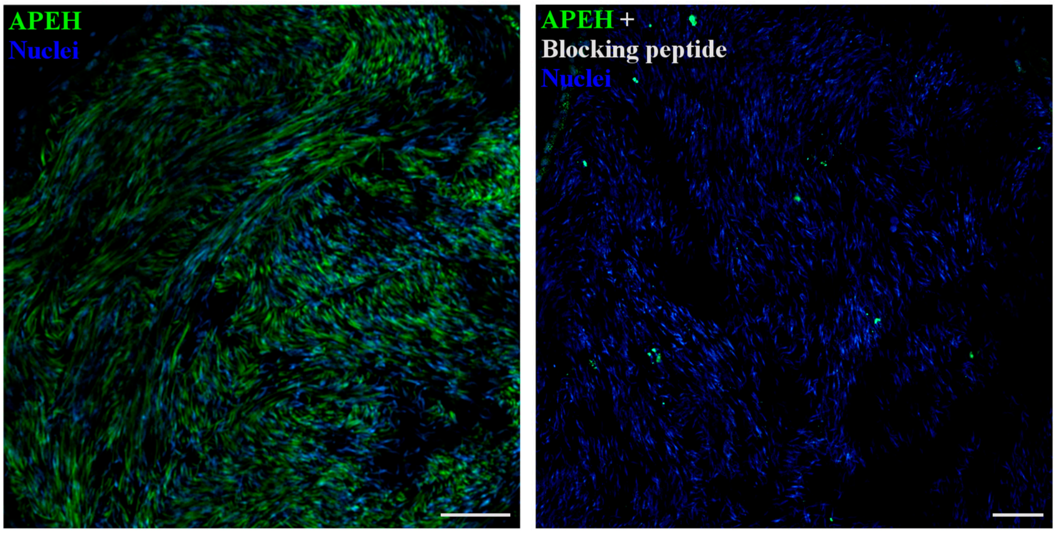

Supplementary figure S2. Peptide competition control against APEH in rat epididymis. (A) Epididymis cryosections were subjected to immunofluorescence against APEH (green). (B) Same as (A), but in the presence blocking peptide LS-E17431 (LSbio). Nuclei were stained with Hoechst 33342 (blue). Scale bar = 50 µm.

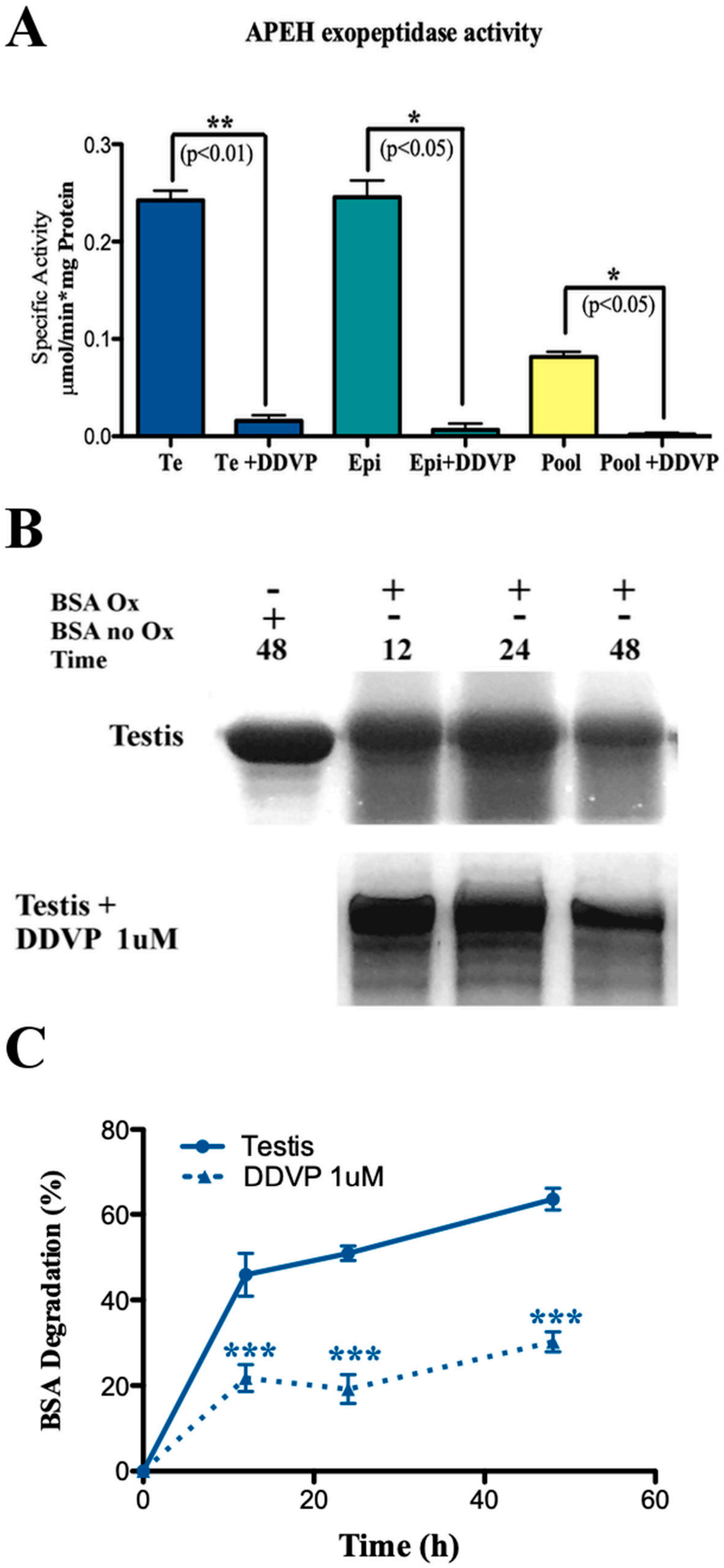
A
APEH exopeptidase activity
Specific Activity
µmol/min*mg Protein
0.3
0.2
0.1
0.0
**
(p<0.01)
*
(p<0.05)
*
(p<0.05)
Te
Te +DDVP
Epi
Epi+DDVP
Pool
Pool +DDVP
B
BSA Ox
BSA no Ox
Time
-
+
48
+
-
12
+
-
24
+
-
48
Testis
Testis +
DDVP 1uM
C
Testis
DDVP 1uM
BSA Degradation (%)
80
60
40
20
0
***
***
***
0
20
40
60
Time (h)

Supplementary figure S3. Inhibition of APEH activity in reproductive tissues and isolated cells. (A) Protein extracts from testis (Te), epididymis (Epi), and pooled germ cells (Pool) were obtained from male rats. Exopeptidase activity was measured at 37°C using N-acetyl-L-alanine p-nitroanilide (AANA) as substrate and results were expressed as specific activity. Protein extracts were incubated with 1 µM Dichlorvos (DDVP) for 1 h at room temperature. Results are representative of three independent experiments. (B) Endopeptidase activity was measured in testis by SDS-PAGE analysis of oxidized BSA incubated at 37°C with testis APEH in the presence or absence of 1 µM DDVP during 12, 24 and 48 hours. (C) Electrophoretic data expressed as BSA degradation percentage at the indicated incubation times versus time 0 obtained by densitometric analysis with Image J software. The results are representative of three independent experiments. Significant differences were found with 2-way ANOVA ($p<0.001$).
